# Depressed mood as a transdiagnostic target relevant to anxiety and/or psychosis: a scoping review

**DOI:** 10.1136/bmjopen-2024-092233

**Published:** 2025-11-24

**Authors:** Edwin Mavindidze, Jermaine Dambi, Primrose Nyamayaro, Rhulani Beji-Chauke, Tariro Dee Tunduwani, Beatrice K Shava, Webster Mavhu, Melanie Abas, Dixon Chibanda, Clement Nhunzvi

**Affiliations:** 1 Department of Rehabilitation Sciences, University of Zimbabwe Faculty of Medicine and Health Sciences, Harare, Zimbabwe; 2 Friendship Bench, Harare, Zimbabwe; 3 Department of Mental Health, University of Zimbabwe Faculty of Medicine and Health Sciences, Harare, Zimbabwe; 4 CeSHHAR Zimbabwe, Harare, Zimbabwe; 5 Liverpool School of Tropical Medicine, Liverpool, UK; 6 Health Service and Population Research Department, King’s College London, London, UK; 7 London School of Hygiene and Tropical Medicine, London, UK; 8 Southern Cross University, Gold Coast, Queensland, Australia

**Keywords:** Depression & mood disorders, Anxiety disorders, MENTAL HEALTH

## Abstract

**Abstract:**

**Introduction:**

Depressed mood is a psychological state characterised by sadness or loss of interest in activities, is a common symptom that accompanies most major mental disorders. It is therefore reasonable to consider it as a transdiagnostic target, which when addressed, may improve the functioning and quality of life of persons with lived experience of mental disorders. However, there is limited understanding of the depressed mood as a transdiagnostic target across major mental disorders. Therefore, this scoping review aims to synthesise knowledge on depressed mood, its measurement and interventions among persons with anxiety and/or psychosis.

**Methods and analysis:**

This scoping review followed Arksey and O'Malley’s framework. Peer-reviewed articles and grey literature published from January 1988 to April 2024 were searched in the following databases: Medline/PubMed, Scopus, Web of Science, Africa-Wide Information, Cumulative Index to Nursing and Allied Health Literature, PsycINFO, SocINDEX, Humanities International Complete, Sabinet, Open Grey and Google Scholar. Articles were screened at title, abstract and full article levels. Data extracted were analysed using thematic analysis and reported following Preferred Reporting Items for Systematic reviews and Meta-Analyses extension for Scoping Reviews guidelines. We also consulted stakeholders such as lived experience experts, clinicians and researchers to contextualise our findings.

**Results:**

We screened 245 full articles out of the 4039 hits and included 28 articles in this review. Although depressed mood is conceptually different from clinical depression, the terms are used interchangeably in the literature. The prevalence of depressed mood in psychosis was 7.3–33.3%, with no prevalence studies specific to anxiety disorders. Commonly used outcome measures included Beck’s Depression Inventory (n=6) and Patient Health Questionnaire-9 (n=5). Psychosocial conservative interventions such as cognitive–behavioural therapy were the most common interventions. Other interventions, including yoga, pharmacotherapy and Ecology Momentary Interventions, were also reported. All interventions were reported to improve depressed mood, and most were implemented in high-income settings. Stakeholders, including lived experience experts, concurred on the importance of using depressed mood as a transdiagnostic target, viewing it as a ‘window’ for early identification and management of many common mental disorders.

**Discussion:**

There is a need to clarify the definition and diagnostic cut-off points on common outcome measures of depressed mood. There is also a need for increased research on depressed mood as a viable transdiagnostic target in anxiety and/or psychosis with a special focus on low-to-middle income countries.

**Conclusion:**

Depressed mood is an important and prevalent transdiagnostic target with great promise for early management in anxiety and/or psychosis. Valid diagnostic and measurement tools are developing, and so are the targeted interventions in the context of anxiety and/or psychosis.

**STRENGTHS AND LIMITATIONS OF THIS STUDY**

- The scoping review methodology is a robust methodology to not only map the available literature, but also to synthesise and identify gaps in the available evidence.
- A clear and replicable research process, including screening and evidence synthesis.
- Involvement of persons with lived experience of depressed mood and other key stakeholders.
- Possibility of language bias as we included only papers published in English.
- Confounding variables were introduced in some of the included studies, in which anxiety and/or psychosis were comorbid presentations in other conditions that could also affect depressed mood.

## Introduction

 Depressed mood is a common early sign and symptom present in most major mental disorders, including mood disorders, psychosis, anxiety disorders, substance use disorders and personality disorders.^1^,^3^ Despite a lack of definitional clarity, depressed mood is largely agreed to refer to a temporal psychological state associated with sadness, irritability, emptiness and/or mild anhedonia.^1 2^ However, it is often used interchangeably with depression.

Individuals with anxiety disorders are 9 times (95% CI 6.3 to 14.3) more likely to also develop depressive symptoms and up to 12 times (95% CI 5.2 to 26.3) in generalised anxiety disorders.^1^ In schizophrenia, the prevalence of comorbid depressed mood can range from 4.6% to 65.1% and is often higher in institutionalised patients than in community-dwelling individuals.^4^ Transdiagnostic interventions may provide an efficient and cost-effective approach to targeting co-occurring symptoms and addressing common presentations in mental disorders.

Transdiagnostic approaches to mental health have gained greater attention in recent years because of growing evidence.^5^ Transdiagnostic interventions are applicable across multiple diagnoses, targeting presenting symptoms without an explicit need for diagnostically aligned treatment protocols, making them efficient, cost-effective and practicable.^6^ Furthermore, transdiagnostic approaches have been advocated because of consensus on the complex nature of mental illness, high comorbidity and the positive response of treatment across diagnostic criteria.^5^ These characteristics are of paramount importance, especially in low-resource settings, which experience a larger treatment gap in mental health.^7 8^ In low-to-middle-income countries (LMICs), disparities between available human and financial resources, and the need for mental healthcare,^8^ call for strengthening evidence of cost-effective approaches that are available at the primary healthcare level and address a wider range of clinical presentations.^9^

The growing recognition of the heterogenous presentation of mental disorders^5^ and increasing evidence of the utility and benefits of transdiagnostic interventions^5 6 10^ calls for reflection on which approaches work and in what conditions. In this light, Wellcome Trust has called for refocusing on co-occurring symptoms and experiences across diagnoses, identified as disabling by persons with lived experience and that may be ameliorated using available treatment strategies.^11^ Focussing interventions on these ‘transdiagnostic targets’ (p.1) has the potential for a more client-centred and practical approach to addressing the real experiences of persons with mental health disorders resulting in improved health-related quality of life and functioning.^11^

Depressed mood is a potential transdiagnostic target across depression, anxiety and psychosis that has been prioritised by persons with lived experience, which may be measurable, modified by available treatment approaches and may lead to an amelioration of functioning. In this paper, we conceptualise depressed mood as a temporary state of lowered mood, sadness, low self-esteem, increased self-criticism and feelings of hopelessness, worthlessness, mild anhedonia and helplessness that do not result in clinically significant distress/dysfunction nor fulfil current clinical depression diagnostic criteria.^12 13^ Furthermore, we conceptualise that individuals with depressed mood score subthreshold scores on depression outcome measures and screening tools. In contrast, depression is a significant lowering of mood and loss of pleasure in all activities markedly differing from previous functioning, which tends to be persistent (≥2 weeks) and results in clinically significant distress and impairments in areas of functioning.^14^ However, it is noteworthy that there has been little focus on depressed mood as a transdiagnostic target, including its prevalence in other major mental disorders like anxiety and psychosis. In addition, there is limited understanding of outcome measures of depressed mood as well as available interventions that improve mood in anxiety and psychosis.

This scoping review, therefore, sought to synthesise the available evidence on depressed mood, and in particular, its epidemiology in psychosis and/or anxiety, outcome measures and potential intervention approaches. Furthermore, we report the insights of persons with lived experience, mental health practitioners and researchers to contextualise our findings and outline possible opportunities for the utility of depressed mood as a transdiagnostic target among persons with anxiety and/or psychosis in LMICs.

## Methods

The scoping review was conducted guided by the six stages of the Arksey and O’Malley framework.^15^ The full methodology is outlined in our previously published protocol.^12^ The purpose of the scoping review was to synthesise evidence on depressed mood as a transdiagnostic target among persons with anxiety and/or psychosis. After an extensive and iterative consultation process with various stakeholders to refine the research questions, including persons with lived experience of anxiety and/or psychosis, the scoping review sought to answer the following:

1. What is the epidemiology of depressed mood in persons with anxiety and/or psychosis?
2. What are commonly used outcome measures for depressed mood?
3. What approaches and interventions are used to modify depressed mood?

### Search strategy

Two researchers (EM and JD) conducted broad searches, guided by the key search terms, on the following electronic databases: PubMed, Scopus, Academic Search Premier, the Cumulative Index to Nursing and Allied Health Literature, Africa-Wide Information, Humanities International Complete, Web of Science, PsycINFO, SocINDEX, Open Grey, Sabinet and Google Scholar. A preliminary search, with the guidance of a subject librarian, was conducted on PubMed and used to refine the search terms and identify MeSH terms. Key search terms included “depressed mood” and its variants, “transdiagnostic” and “psychosis” and its variants, and “anxiety” and its alternative terms (table 1). The search terms were combined using various Boolean commands to glean the literature. The search strategy was adapted accordingly for all the databases. We initially searched for literature published between January 2004 and April 2024. The year 2004 was selected as the start date because quick pilot searches showed it to be the year the first article on transdiagnostic targets was published.^16^ We also reviewed the references of included articles for relevant articles we may have missed in our search. We then revised our search dates to 1988, as our initial readings suggested that though the term ‘transdiagnostic’ was not used in earlier research, the concept existed in studies of depressed mood in psychosis and/or anxiety.

**Table 1:** Key terms for database search

Key search terms	Alternative terms	PubMed
Depressed mood	Depressive symptoms OR Depressive affect OR Depress*	Depression (MeSH)
Transdiagnostic		Transdiagnostic
Psychosis	Psychotic disorder OR Psychoses OR Psycho* OR Schizophreni* (For schizophreniform, Schizophrenia) Schizoaffective OR Brief reactive psychosis OR Substance-induced psychoses	Psychotic Disorders (MeSH) OR Psychoses OR Substance-induced (MeSH)
Anxiety	Social anxiety OR Nervousness OR Anxiousness OR Generalised anxiety disorder OR Panic attack OR phobia Anxiety Disorder OR Anxi* OR Neurotic OR Neuroses	Anxiety (MeSH) OR Anxiety Disorders (MeSH)

### Study selection

After the literature search, results were uploaded to Rayyan^17^ to prepare for the screening process. Two review teams (Team 1: JD and PN; Team 2: RB-C and EM) screened the identified articles at title, abstract and full article levels. The teams first independently reviewed the identified articles by title and abstract on Rayyan against predetermined selection criteria (table 2). The potential articles were captured by the two teams, and the lists were compared. In cases of reviewers’ disagreement(s), a consensus meeting was convened, where the article was further reviewed, and a decision was agreed on. If there was uncertainty, the article was further reviewed in the full article screening step. Full texts of selected articles were retrieved, and two reviewers not involved in the title and abstract screening (CN and WM) read through the full article for further screening and captured it on an Excel spreadsheet with a recommendation to include or exclude. The spreadsheets from the two teams were compared and disagreements settled by consensus. Throughout all three steps, Cohen’s kappa was calculated to assess the level of agreement between reviewers. If the level of agreement was weak (κ<0.60), the process was repeated until there was a strong agreement. Included articles were separated in preparation for data extraction.

**Table 2:** Selection criteria for articles

Study inclusion criteria	Study exclusion criteria
Peer-reviewed articles, grey literature, original research and conceptual papers. Literature published in English. Date range (January 1988–April 2024). Literature on depressed mood in patients with anxiety and/or psychotic disorders per Diagnostic and Statistical Manual fifth edition.	Subthreshold anxiety. Clinical depression.

### Data extraction and synthesis

The research team developed a data extraction form guided by a review of similar studies. The form went through iterative review and consultation among the team members, and a panel of stakeholders also reviewed it for contextual relevance. The data extraction form was applied to a small sample of articles before making final refinements. Bibliometric information including the authors, year of publication, country of study, the study design and study population, together with results relevant to the scoping review, was captured on an Excel spreadsheet. Using inductive thematic analysis, we summarise the outcome measures and interventions for transdiagnostic depressed mood in anxiety and/or psychosis. We present our findings in a typical narrative format with a tabular supplement. We also present charts highlighting the distribution of included studies by geography, year, diagnosis and nature of study. Our reporting is guided by the Preferred Reporting Items for Systematic reviews and Meta-Analyses extension for Scoping Reviews checklist.

### Consultation exercise

In line with Arksey and O’Malley’s^15^ sixth stage, we again consulted stakeholders on our results. Applying principles of research co-creation and a collaborative approach, we engaged three persons with lived experience, as strategic stakeholders, experts by experience, reviewers and knowledgeable partners, throughout the review process. We triangulated ways of involvement by engaging them in an expert advisory committee, as key informants, research assistants and process and outcome reviewers. In all our engagements, we were guided by principles of knowledge inclusion, diversity and equality.

To gain more insights into the results and understand the transdiagnostic utility of depressed mood in anxiety and/or psychosis, we critically discussed our preliminary findings with various stakeholders, including persons with lived experience, clinicians and researchers (table 3). We recorded, transcribed and thematically analysed these discussions. We also conducted these consultative discussions to check if we had not missed any key resources and articles in our search and to contextualise our findings. We present some of their insights, thereby triangulating them with review findings.

**Table 3:** Stakeholder profiles

Pseudonym	Stakeholder profile
Clive	Male; lived experience expert; recovering from psychosis
Farayi	Female; lived experience expert; recovering from anxiety disorder
Tsitsi	Female; lived experience expert; recovering from psychosis
Peter	Male; mental health researcher
Rose	Female; occupational therapist in mental health
Hope	Female; psychiatrist
George	Male; psychiatric nurse
Rufaro	Female; clinical psychologist

## Results

### Bibliometric characteristics

**Figure 1:**
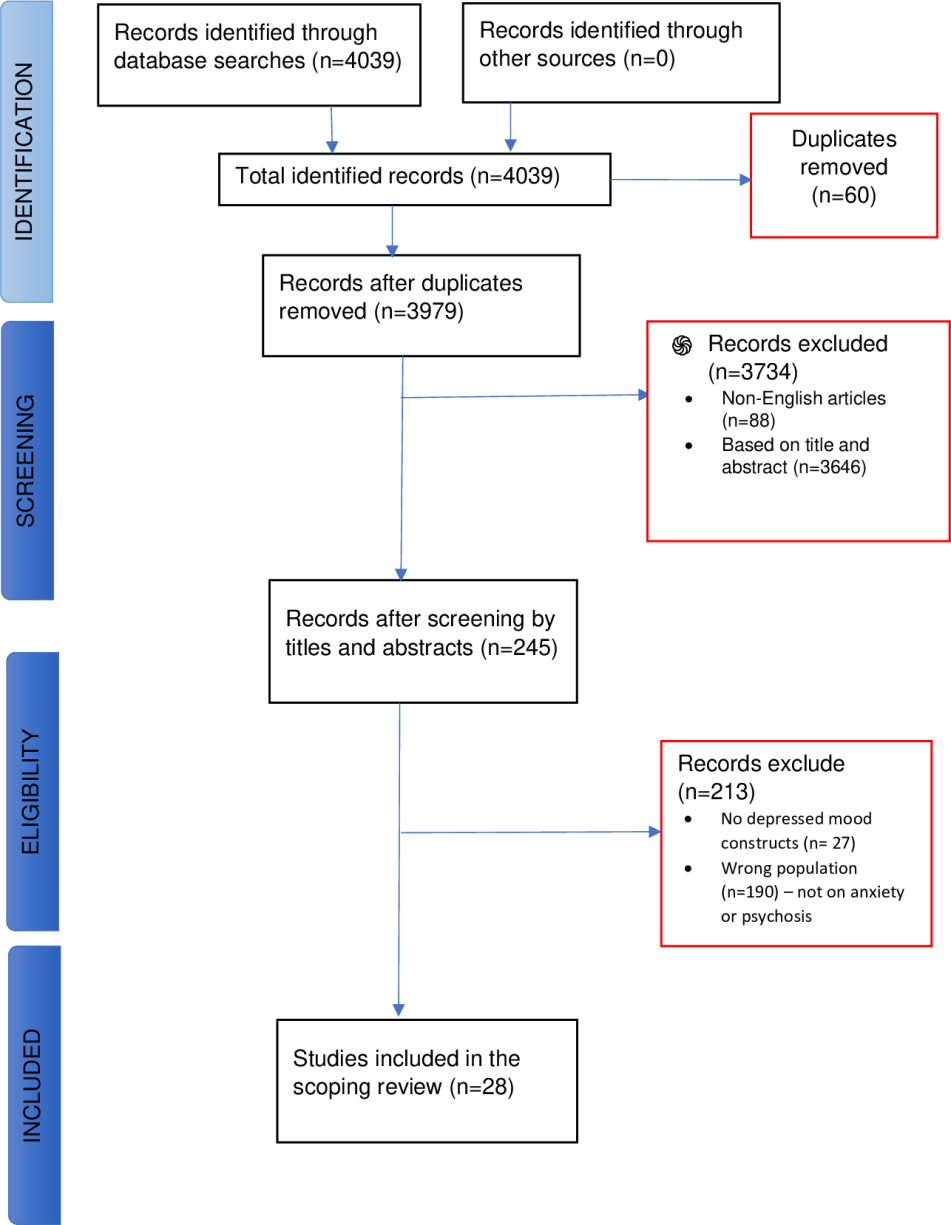
PRISMA chart for the scoping review. PRISMA, Preferred Reporting Items for Systematic Reviews and Meta-Analyses.

The search and screening yielded 28 articles. Figure 1 included studies were published between 1988 and 2023. Five studies were published in 2023, three in 2022 and two studies apiece published in the years 2003, 2005, 2007, 2014 and 2021. Eight studies were conducted in the USA, with others coming from the UK (n=5), the Netherlands (n=3), Mainland China (n=3), Italy (n=2) and Ireland (n=2). A single article each reported findings from Iran, Germany, Canada, India and Australia.

The analysed studies reported on individuals with anxiety disorders (n=8), psychoses (n=9), pregnant women (n=3), individuals at risk of type-2 diabetes (n=2), students (n=1), epilepsy (n=1), adults with obesity (n=1), adults with low mood (n=1), women with polycystic ovarian syndrome (n=1), tinnitus sufferers (n=1) and the general population (n=1). Study designs were randomised-controlled trials (n=8), cross-sectional studies (n=5), longitudinal/cohort designs (n=8), quasi-experimental designs (n=3), case-control designs (n=2), case series (n=1) and clinical audit (n=1) (see online supplemental table S1 for the characteristics of included studies).

### Prevalence of depressed mood in anxiety/psychosis

The terms depression and depressed mood were used interchangeably in literature.^18^ None of the reviewed articles reported on the prevalence of depressed mood in anxiety disorders, despite an established knowledge base around the intricate relationship between depression and anxiety.^19^,^21^ However, included articles reported a prevalence of depressed mood in psychoses of 7.3–33.3%.^22^,^24^

### Common outcome measures for depressed mood

Thirteen outcome measures were reported. Of these, the Beck Depression Inventory (BDI) (n=6)^25^,^30^ and the Patient Health Questionnaire-9 (PHQ-9) (n=5)^30^,^34^ were the most used depressed mood outcome measures. Other outcome measures included the Positive and Negative Syndrome Scale (PANSS) (n=4),^22^,^2435^ Hamilton Depression Scale (HDS) (n=3),^36^,^38^ Depression, Anxiety and Stress Scale (DASS) (n=3)^16 39 40^ and Hospital Anxiety Depression Scale (HADS) (n=2).^41 42^ The analysed outcome measures were depression-specific measures (eg, BDI, PHQ-9, HDS and Montgomery-Asberg Depression Scale (MADRS)) and generic depressed mood outcome measures (eg, DASS, HADS, PANSS, Mood and Anxiety Symptom Questionnaire (MASQ) and Exercise and Health Questionnaire). Also, many of the studies reviewed only stated the outcome measures that were used to assess depressed mood without describing the characteristics of the outcome measure or the rationale for its selection. However, some provided a basic description of the BDI, PHQ-9, HADS and PANSS.^2224 29 31^,^33 41^ The BDI is a commonly used 21-item validated measure of depression symptoms, the PHQ-9 is a 9-item questionnaire that screens for depressed mood and symptoms of depression in the past 2 weeks and the HADS is a 14-item measure of anxiety and depression used for the general population.^29 31 41^ All three are self-administered and have been reported as highly reliable with internal consistencies ranging from Cronbach α=0.84 to α=0.94.^30 31 33 34 41^ The PANSS only has a single item that measures depressed mood.^22^ Wei *et al*^31^ reported that a local language (Chinese) version of the PHQ-9 was available and they used it in their study.

### Approaches and interventions to modify depressed mood

Our review demonstrates tentative evidence regarding the positive effect of interventions delivered to people experiencing psychosis and anxiety on depressed mood. Psychosocial interventions for psychosis and anxiety, even without the use of depression-specific techniques, improved depressed mood.^16^ The most reported intervention was cognitive–behavioural therapy (CBT), which was mainly applied in a group format.^16 27 28 38 42 43^ However, most of the studies do not report on the actual CBT techniques that were applied, limiting the identification of the active ingredients to modify depressed mood. Only Norton *et al*^16^ reported that they did not apply depression-specific CBT techniques, such as behavioural activation, challenging automatic thoughts or scheduling pleasant events, but still recorded an improvement in depressed mood. Other psychological interventions, including Cognitive Therapy for Social Anxiety Disorder^30^ and Group-based self-esteem programme^26^ were also reported to have a positive outcome on depressed mood. Yoga,^41 44^ pharmacotherapy^40^ and Ecology Momentary Interventions (EMI)^29^ were also reported to improve depressed mood in anxiety and/or psychosis. Interventions were combined with physical exercises and nutrition advice in some population groups.^42^

### Reflections and insights from stakeholders

After reviewing our findings, stakeholders seconded that depressed mood was a prevalent and emerging transdiagnostic target of interest in anxiety and/or psychosis, with a developing evidence base. Despite the interchangeable use with depression and a lack of definitional clarity, stakeholders saw depressed mood as a ‘window’ through which early identification and effective management of a number of mental disorders could be initiated. Depressed mood was conceptualised as a tool, through which one sees beyond traditional diagnostic boundaries and an opportunity to explore beyond the presenting concern, be it anxiety or psychosis. One of the stakeholders succinctly reflected on this and said:

Depressed mood is a ‘window’ through which we view both the inside and the outside realm of either psychosis or anxiety… through it (depressed mood), in-depth issues can be explored and addressed…, to focus on it would be very strategic for early intervention and better outcomes… (Peter, mental health researcher).

Stakeholders also provided insights into the implications of the review findings regarding understanding depressed mood as a transdiagnostic target in both anxiety and psychosis. We present these insights in three themes: the lack of definitional clarity and the role of lived experience; diagnosis and measurement; and the intervention gap.

### The lack of definitional clarity and the role of lived experience

Without a clear working definition of depressed mood, its utility is challenged and there are also cultural influences such as masculinity at play. Despite it being a common precursor and prevalent in persons with anxiety and/or psychosis, both the lived experience experts and clinicians concurred that there was a challenge regarding the fluidity of depressed mood definition and its conceptualisation. In most cases, a depressed mood was taken to mean a mild form of depression or the very early signs of depression, anxiety or psychosis emphasising the presence of sad mood.

In some cultures, a depressed mood could be played down as part of normal reaction to stress. These cultural practices, coupled with low mental health literacy and poor health-seeking behaviours, might result in individuals not seeking timely professional services, leading to the outcome’s worsening. One of the lived experience experts went on to highlight the normative and masculinity cultural practices implicated:

… before my first episode of psychosis, I knew I was abnormally sad and had been feeling low for some time, but never sought help… in reflection I see that was depressed mood, right? But here as a man, you are told you shouldn’t be a ‘cry-baby’*…* (Clive, male; lived experience expert).

Asked about the scoping review finding that depressed mood was often confused with depression and vice versa, the stakeholders reiterated the need to depart from diagnostic labels and rather attend to the functional challenges the person will be having, considering contextual realities, including how depressed mood was affecting self-care and productive life participation.

### Diagnosis and measurement

Early screening, detection and management of depressed mood may be influenced by how it is diagnosed and assessed. The importance of early screening and detection of depressed mood was emphasised as a key factor in managing the condition effectively.

Stakeholders agreed on the need for the development and validation of standardised tools, as these would set treatment protocols or guidelines in motion. However, standardised tools alone may not give a fuller picture without service user narratives of how they experience depressed mood. It was stated that both diagnostic criteria and the voice of the person experiencing the condition should be considered in understanding and addressing depressed mood. The insights shared spoke to the limitations of diagnostic criteria and the importance of hearing the experiences of individuals, especially in cases where symptoms may be withheld or difficult to express.

The clinicians highlighted the lack of specific tools for depressed mood and mentioned that depression tools were serving the purpose, though one needs more clinical reasoning to differentiate depressed mood from depression.

…depression screening scales and diagnostic tools including PHQ-9, SSQ-14, ICD-11 and DSM are what we currently use … of course not enough as you still have to get it from the person’s own story… (Hope, psychiatrist).

### The intervention gap

Conceptualisation issues and measurement challenges are associated with intervention gaps. Transdiagnostic interventions for depressed mood in anxiety and/or psychosis would target the shared mechanisms underlying the condition. Lived experience experts and other stakeholders agreed that depressed mood was a common factor in both anxiety and psychosis, which could benefit from the same interventions used in the management of depression, with more inclination towards the non-pharmacological therapies.

… we can agree that depressed mood can best be managed using more supportive care, … psychosocial therapies, more like treating early and mild forms of depression… (Rufaro, clinical psychologist).

Clinicians, researchers and academics questioned the practicalities of managing depressed mood as a singular transdiagnostic target without considering the complexities of anxiety and/or psychosis. They, however, suggested the need for both early intervention and stratification of intervention intensity and complexity based on symptom presentation and general person needs. Nevertheless, the lived experience experts posited that they would rather have their depressed mood targeted first in interventions as it turned to have ripple improvements in other domains.

… on reflection, I would say I want to have my depressed mood attended to early, because that way, I can effectively benefit from other treatments, … even motivation to try, participate and take my medication… (Tsitsi, female; lived experience expert).

## Discussion

This study sought to synthesise findings from the literature and key stakeholders’ perspectives regarding knowledge on depressed mood as a transdiagnostic target among persons with anxiety and/or psychosis. The interchangeable use of the terms ‘depression’ and ‘depressed mood’ was a key finding. Also, most studies were conducted in high-income countries, and the PHQ-9 was the most applied outcome measure, with some reasonable evidence supporting the use of CBT in managing depressed mood. None of the studies explicitly highlighted any advantages or otherwise of using any of the outcome measures they used to assess depressed mood. The subsequent sections discuss the implications of the review.

Although depressed mood is conceptually different from clinical depression,^45 46^ the terms are used interchangeably in the literature. Parker and Paterson^47^ suggest that challenges in this differentiation are based on limitations in the distinguishing parameters of clinical and non-clinical presentation of depressive symptoms. For instance, there are inconsistencies among studies in defining depression, varying between a formal diagnosis and defining it in terms of dysphoria/depressed mood.^48^ Whereas depression is succinctly defined as a loss of pleasure or interest in activities, and associated classical symptoms (eg, loss of concentration, disrupted sleep) per Diagnostic and Statistical Manual of Mental Disorders or International Classification of Disease criteria,^45 46^ no such standardised clinical diagnostic criteria exist for depressed mood.^47^ Also, depression is diagnosed on the frequency and duration of symptoms persistence.^45 46^ Again, there is no corresponding criteria for depressed mood, further compounding the ambiguity in the use of the two terms. It is therefore unsurprising that depressed mood is frequently misinterpreted for various presentations and symptoms of depression.

A term frequently interchanged with depressed mood is subthreshold depression, which may be defined as presenting with four or less depressive symptoms or diagnostic criteria in 2 weeks with an associated social dysfunction.^49^ However, while acknowledging that there is no universal definition of subthreshold depression because of a heterogeneity of conceptualisations, Rodriguez *et al*^49^ make a clear distinction between depressed mood and subthreshold depression, highlighting that although it is often an accompanying symptom, some definitions exclude the former entirely from the criteria of the latter. Therefore, while subthreshold depression may be conceptualised on a severity continuum with major depressive disorder, where the full diagnostic criteria is unmet over the 2-week period; depressed mood represents a group of symptoms which may or may not accompany a pathological depressive presentation and is often transient. Also closely related to depressed mood is the concept of anhedonia. Anhedonia denotes a lack of interest in activities that were previously pleasurable to the individual.^50^,^52^ However, depressed mood and anhedonia, though closely related, are not interchangeable terms. Although anhedonia is also a transdiagnostic symptom across many mental disorders,^51^ it only characterises depressed mood when it is transient and does not constitute a clinically significant pathology but is not in itself depressed mood. Similarly, emotional blunting—an ‘inability to feel positive or negative emotions; feelings of detachment; or reduced emotional responsiveness’^53^—is another transdiagnostic feature in psychosis and depressive disorders. From its definition, it is contrary to depressed mood characterised by negative emotions such as sadness, irritability and hopelessness, which are lacking in emotional blunting.^54^ Furthermore, emotional blunting is a common side effect of the antidepressant medications used to treat depressed mood.^53 54^

Future studies should seek to differentiate the conceptualisation of depressed mood and subthreshold depression. Without clarifying these terms and their conceptualisations, it may lead to overlapping constructs and outcomes that are difficult to measure, affecting their clinical utility and synthesis of evidence. There is a need for further conceptualisation of depressed mood if it is to be used as a transdiagnostic target clinically, in research or policy formulation. Given the need for standardised nomenclature, we propose a working definition for depressed mood based on previous studies and our stakeholders’ consultations. We operationalise depressed mood as a temporary psychological state with affected individuals expressing at least one of the classical depressive symptoms such as sadness, irritability, emptiness, unhappiness or loss of pleasure/interest in activities.^1 2^ However, the symptoms are not persistent, with minimal functional impacts and may be viewed as a transitional state to the evolution of other mental conditions if untreated or if there is exacerbation of the causative agents.

Although not universally conceptualised, evidence from the few studies analysed suggests the highly variable prevalence of depressed mood across diagnosis and contexts. The variability was more pronounced in those with psychosis, ranging from 7% to as high as 33%. Several contextual factors were cited which might explain this variability. The context of the study population may have a profound effect on the prevalence of depressed mood,^22^ including a potentially protective hospital environment that provided a therapeutic milieu for chronically hospitalised patients with psychosis. Conversely, Tan *et al*^55^ reported a higher prevalence because they studied patients in a military hospital who also recorded an even higher prevalence of depressed mood among patients without psychosis, suggesting the influences of past experiences related to military engagement and associations with post-traumatic stress disorder.

Our review highlights the need for more research from LMICs to ensure a more representative understanding of the epidemiology of depressed mood in anxiety and psychosis. Additionally, this has the potential to amplify the use of depressed mood as a transdiagnostic target across contexts and explore possible cultural influences. Also, further research is needed to better understand the prevalence of depressed mood in anxiety and to explore potential factors contributing to its variability in psychoses.

We retrieved several depressed mood outcome measures; the same tools used for screening and diagnosing depression. The interchangeable use of outcome measures reflects the lack of clarity in the definition of depressed mood. Of 13 outcome measures found, the BDI and the PHQ-9 were the most commonly used measures. This is not surprising as both measures have been revised and structured around the diagnostic criteria and symptomatology of depression, are available free of charge and have extensive transcultural validity.^56 57^ The scales used only differentiate depressed mood from depression on the diagnostic threshold and not necessarily by item measure. For instance, the PHQ-9 was specifically designed to measure depression at a cut-off score of ≥10 and a score of 5–9 signifying mild depression.^58^,^60^ A score of ≤4 may suggest the presence of depressed mood but is considered insufficient for a diagnosis of depression.^61 62^ Furthermore, it should be noted that the various outcome measures reported use various scales and ratings for depressed mood. While outcome measures for psychosis like the PANSS have a single item for depressed mood, this does not have the same psychometric robustness for measuring depressed mood as would the PHQ-9 or BDI, with the latter requiring a score of at least 11 on multiple items to suggest presence of depressed mood. Consequently, there is a need to apply reflective measurement models^63^ to develop bespoke depressed mood outcome measures. Given the overlap with depression, future studies should consider leveraging existing depression measures but adapt the items to increase the tools’ discriminative abilities. Consequent, depressed mood outcome measures should explicitly define the recall period, system intensity and duration to clearly capture depressed mood as a distinct construct.

We found empirical evidence supporting the potential impact of a range of interventions, including CBT, yoga, pharmacotherapy, exercise, nutrition and EMI, to modify depressed mood effectively. The common factor among the approaches and interventions that positively modify depressed mood is their focus on positively changing thought content. Cognitive therapy-based interventions are primarily based on the identification of maladaptive thoughts and developing cognitive behavioural strategies to overcome these thoughts.^27 28 38^ On the other hand, EMI is a psychological intervention that prompts the individual to direct their focus towards positive thoughts and memories and the positive emotions associated with these thoughts.^29^ The exact content of the cognitive-based intervention is determined by the primary purpose of its use. For example, for generalised anxiety disorders, CBT includes relaxation techniques like guided imagery and progressive muscle relaxation,^28^ while it might also include techniques based on exposure to feared situations.^38^ Befittingly, transdiagnostic interventions like CBT^6^ could show positive outcomes in addressing a transdiagnostic target like depressed mood.

Also, evidence suggested yoga as a viable transdiagnostic intervention for depressed mood. Yoga included the use of posturing, meditation and relaxation techniques for managing depressed mood.^41 44^ Mindfulness improves psychological resilience, enabling individuals to avoid ruminations and negative thinking that characterises the dysfunctional schema of the depression triad.^64^ EMI approaches are also based on mindfulness, explaining how they have a positive effect on depressed mood.^65^,^67^ Lastly, our review showed promising evidence regarding interventions combining physical activity with nutritional advice.^42^ D’Angelantonio *et al*^68^ suggest that physical exercise positively modifies depressed mood. However, it is important to note that individual responses to interventions may vary, and a personalised transdiagnostic approach is often necessary to effectively modify depressed mood.

Of note, most of the interventions highlighted above, although having positive outcomes for depressed mood, were implemented targeting symptoms of anxiety or psychosis. There is a knowledge gap on interventions that specifically target depressed mood, more so in LMICs. This might be because depressed mood is often not considered to warrant intervention.^47 69^ There is a need for research into the prognosis of anxiety and psychosis if depressed mood is targeted, so as to promote its uptake as a transdiagnostic target.

We originally planned to assess the psychometric robustness of the various measures of depressed mood and the effectiveness of the interventions to modify it.^12^ However, due to the nature and quality of the reviewed studies, the available articles could not provide sufficient data to comprehensively answer the questions. We acknowledge that it is important to assess the psychometric properties of the measures and the effectiveness of the interventions to modify depressed mood; we recommend a systematic review with sufficient high-quality data to comprehensively answer both questions.

## Conclusions

This sequential, exploratory mixed review provides preliminary insights into the burden and management of depressed mood in persons with anxiety and psychosis. There is a need to standardise the term ‘depressed mood’ to operationalise its measurement and treatment. To this end, we propose an operational definition of depressed mood to mean temporary psychological state with reference to an individual’s mood expressing at least one of the mild to moderate classical depressive symptoms such as sadness, irritability, emptiness, unhappiness or loss of pleasure/interest in activities, with minimal functional impacts. Needless to say, a depressed mood seems to be highly prevalent in persons with comorbid anxiety and psychosis. There is, therefore, a strong need to develop culturally responsive and targeted transdiagnostic interventions, even more so, that the lived experience experts in this work stated that depressed mood is a viable target that can be used in treatment for other mental health conditions.

## Supplementary material

10.1136/bmjopen-2024-092233 online supplemental file 1

## Data Availability

Data sharing not applicable as no data sets generated and/or analysed for this study.
