# Supplementary material for: Depressed mood as a transdiagnostic target relevant to anxiety and/or psychosis: a scoping review
Source: BMJ Open. 2025 Nov 24;15(11):e092233. doi: 10.1136/bmjopen-2024-092233 (PMC12645650; doi:10.1136/bmjopen-2024-092233)
Supplement: online supplemental file 1 [file bmjopen-15-11-s001.docx]

Table S1: Summary of articles on transdiagnostic depressed mood

|  | **Author [Year]** | **Country** | **Study Design** | **Population** | **Sample size** | **Age [Mean (SD)]**  **SD: Standard Deviation** | **Depressed mood outcome measure** | **Intervention** | **Effect on depressed mood** |
| --- | --- | --- | --- | --- | --- | --- | --- | --- | --- |
| 1 | Langi et al [1988] | Netherlands | Randomised Control Trial (RCT) | Generalized Anxiety Disorder | N = 48 | ●Experimental 40.1 (SD 10.8)  ●Control 36.5 (SD 12.2) | Norris, Bond and Lader Visual analogue mood rating scale | Ritanserin | Positive improvement on depressed mood |
| 2 | Mennin, Heimberg & Jack [2000] | USA | Quasi-experimental | Social phobia and generalised anxiety disorder | N=122 | ●SP with GAD – 35.8 (SD 10.1)  ●SP without GAD= 37.1 (SD 10.2) | Hamilton Depression Scale (HDS) | Cognitive-behavioural Therapy (CBT) | Positive improvement on depressed mood |
| 3 | Evans et al [2003] | USA | Case-Control | Schizophrenia and schizoaffective disorder | Schizophrenia, n=93 & Healthy Individuals, n=73 | Schizophrenia Mean = 57.2 (SD 9.1) & Healthy Individuals Mean = 59.2 (SD 11.2) | HDS | N/A | N/A |
| 4 | Cuthbert et al [2003] | USA | Quasi-experimental | Anxiety disorders | N= 130 | 30-40 years | Beck Depression Inventory (BDI) | Fear memory imagery | None reported |
| 5 | Norton, Hayes & Hope [2004] | USA | RCT | Anxiety disorders | N=23 | mean & SD NR. 19 or older | Depression, Anxiety, And Stress Scales (DASS) | CBT | Positive improvement on depressed mood |
| 6 | Gruettert & Friege [2005] | Germany | Case Series | Borderline personality disorder & Psychosis | N = 12 | 24.6 (SD 4.1) | HDS | Quantiapine | Positive improvement on depressed mood |
| 7 | Rieckmann et al [2005] | USA | Cohort | Schizophrenia patients | N= 657 | 74.2 (SD 11.8) | Positive and Negative Syndrome Scale (PANSS)- (Single item) Depressed mood  Alzheimer’s Disease Assessment Scale – Late Version (ADASL)- (single item) Depressed mood | N/A | N/A |
| 8 | Brébion et al [2007] | UK | Case Control | Schizophrenia | Schizophrenia N= 48 Healthy Individuals N= 41 | Schizophrenia -34.7 SD (7.9)  Healthy Individuals- 35.1 SD (9.9) | Depression – Montgomery and Asberg Depression rating Scale (MADRS) | N/A | N/A |
| 9 | Laithwaite et al [2007] | UK | Quasi-experimental | Schizophrenia & schizo-affective disorder | N=15 | 35.27 (SD 8.8) | BDI | Group-based self-esteem program. | Positive improvement on depressed mood |
| 10 | Fowler et al [2012] | UK | Cohort | Nonaffective psychosis (schizophrenia, schizoaffective disorder & delusional disorder) | N=301 | 37.6 (SD 11.0) | BDI | CBT | Positive improvement on depressed mood |
| 11 | Satyapriya et al [2013] | India | RCT | Pregnant women | N= 96; yoga n = 51, control n =45 | yoga = 26.41 years (SD 3.01)  Control = 24.96 years (SD 2.58) | Hospital Anxiety Depression Scale (HADS) – Anxiety and depression | Yoga | Positive improvement on depressed mood |
| 12 | Queen et al [2014] | USA | RCT | Adolescents with anxiety & depressive symptoms | N=59 | M = 15.42 years (SD = 1.71)  Range =12-17 years | Revised Children’s Anxiety and Depression Scale (RCADS) | Unified Protocol for the Treatment of Emotional Disorders in Adolescence | No significant change |
| 13 | Schlosser et al [2014] | USA | Cross sectional/ Descriptive Analytical | Psychosis: those at clinical high risk (CHR) of developing a psychotic disorder, schizophrenia or schizoaffective disorder. | N = 234 | HC= 17.6 (3.7); CHR = 18.6 (4.6); RO = 21.7 (4.5); SZ = 42.9 (10.9) | PANSS | N/A | N/A |
| 14 | Newham et al [2015] | UK | RCT | primiparous pregnant women | N=59; TAU n = 28, yoga n= 31 | TAU =31(7); yoga = 31(5) | Edinburg Postnatal Depression Scale (EPDS)- Depressive symptoms | Yoga | Positive improvement on depressed mood |
| 15 | Deschenes et al [2016] | Canada | Cohort Study | men and women without diabetes | N= 2486; non-prediabetes n = 1428, prediabetes = 1058 | Range= 40- 70 years | Patient Health Questionnaire -9 (PHQ-9) | N/A | N/A |
| 16 | Kell et al [2017] | Australia | RCT | adults with low mood | N=128 |  | DASS | Saffron | Positive improvement on depressed mood |
| 17 | Deschenes et al [2018] | Netherlands | Cohort Study | adult non-diabetic participants | N = 78025; No D/A = 73483, A = 1910, D = 1634, CAD = 998 |  | Mini International Neuropsychiatric Interview (MINI) | N/A | N/A |
| 18 | Thew et al [2020] | UK | Clinical Audit | Patients with Social Anxiety Disorder | N=271 Sample 1 n=185  Sample 2 n=86 | 32.2 (8.6)  33.2 (9.5) | PHQ-9  BDI | Cognitive therapy for Social Anxiety Disorder | Positive improvement on depressed mood |
| 19 | Wei et al [2021] | China | Cross -sectional | epileptic patients | N=313 | 31.5 +/-(2.3) | PHQ-9 | N/A | N/A |
| 20 | Ren et al [2021] | China | Cross -sectional | nursing students | N=776 | 18.87(.95) | PHQ-9 | N/A | N/A |
| 21 | Herring [2022] | Ireland | Cross-sectional | Generalised anxiety disorder | n= 470 | 23.24±4.81 | Single depressed mood item on the Exercise & Health Questionnaire | N/A | N/A |
| 22 | Mohseni [2022] | Netherlands | Longitudinal study | Adults with obesity | N=96 | 18 | Hospital depression and Anxiety Score (HADS) | CBT | Positive improvement on depressed mood |
| 23 | LaFreniere & Newman [2023] | USA | RCT | Generalized anxiety disorder | N= 85; skilljoy n=41, control n= 44 |  | BDI | EMI | Positive improvement on depressed mood |
| 24 | Chen et al [2023] | China | Cross -sectional | patients with tinnitus | N= 566 | 44.74 (14.33) | PHQ-9 | N/A | N/A |
| 25 | Hannon et al [2023] | Ireland | Longitudinal study | Pregnant women | N=1804 | 18 | DASS | N/A | N/A |
| 26 | Majidzadeh et al [2023] | Iran | RCT | Women with PCOS | N = 84; CBT = 42, control =42 | CBT = 30.3 (5.5); Control 32.0(4.8) | BDI | CBT | Positive improvement on depressed mood |
| 27 | Pelizza et al [ 2022] | Italy | Longitudinal | People aged 12-35 years with first episode Psychosis | N=266 | 24. (20-30) | PANSS | Phamarcotherapy(Antipsychotics and antidepressants)  Individual psychotherapy – cognitive-behavioural modules | Baseline measures |
| 28 | Pelizza et al [2023] | Italy | Longitudinal | Patients aged 12-35 diagnosed with first-episode psychosis | N=159 | 23.29 ± 5.36 years) | PANSS | Pharmacotherapy  Individual psychotherapy | Positive improvement on depressed mood |
