# Supplementary material for: Depressed mood as a transdiagnostic target relevant to anxiety and/or psychosis: a scoping review
Source: BMJ Open. 2025 Nov 24;15(11):e092233. doi: 10.1136/bmjopen-2024-092233 (PMC12645650; doi:10.1136/bmjopen-2024-092233)
Supplement: Supplementary file 2 [file bmjopen-2024-092233.reviewer_comments.pdf]

# PEER REVIEW HISTORY

BMJ Open publishes all reviews undertaken for accepted manuscripts. Reviewers are asked to complete a checklist review form and are provided with free text boxes to elaborate on their assessment. These free text comments are reproduced below.

## ARTICLE DETAILS

### Title (Provisional)

Depressed mood as a transdiagnostic target relevant to anxiety and/or psychosis: a scoping review

### Authors

Mavindidze, Edwin; Dambi, Jermaine; Nyamayaro, Primrose; Beji-Chauke, Rhulani; Tunduwani, Tariro Dee; Shava, Beatrice K.; Mavhu, Webster; Abas, Melanie; Chibanda, Dixon; Nhunzvi, Clement

---

## VERSION 1 - REVIEW

---

| | |
|---|---|
| **Reviewer** | **1** |
| **Name** | **Subramaniam, Mythily** |
| **Affiliation** | **Institute of Mental Health, Research** |
| **Date** | **11-Dec-2024** |
| **COI** | **None** |

---

The authors have conducted a scoping review to synthesise knowledge on depressed mood, its measurement, and interventions among persons with anxiety and/or

psychosis.

The article is well written and the methodology of the review is stringent. I have some concerns that I would like the authors to address.

1. The authors mention that to meet criteria for depressed mood the participants should not have clinically significant distress/dysfunction. However, they make no mention of how this was assessed in the studies included in the review or in their discussion.

2. How was the data extraction form developed and was it trialed in any way?

3. While not part of their scoping review they should discuss the concept of sub-threshold depression, which in many ways is better defined and how do they think depressed mood is different from it and the need to focus on depressed mood. Otherwise, we have various terminologies being used which are both overlapping and confusing.

4. Was any ethical approval or consent sought for the inclusion of the panel comprising experts and those with a lived experience?

---

| | |
|---|---|
| **Reviewer** | **2** |
| **Name** | **pitanupong, jarurin** |
| **Affiliation** | **Prince of Songkla University, psychiatric** |
| **Date** | **19-Jan-2025** |
| **COI** | **None** |

---

Thank you for allowing me to review this manuscript. I believe that this is good research that pays attention to the depression or subthreshold depression that may affect the quality of life of patients with anxiety and psychosis. However, nowadays there is the word " anhedonia, or emotional bunting, is one of the more common depressive symptoms that may not have been included in the study. I want the author to discuss this in detail.

In addition, I would like the author to check the English language, including the uppercase or lowercase letters used in some points that may not be appropriate.

Thanks, and good luck.

---

| | |
|---|---|
| **Reviewer** | **3** |
| **Name** | **Black, Melissa** |
| **Affiliation** | **MRC Cognition and Brain Sciences Unit, University of Cambridge** |
| **Date** | **01-Sep-2025** |
| **COI** | **None** |

---

Thank you for the opportunity to review this interesting paper, it is well-presented and outlines a thought-provoking scoping review. The rationale for the review is clear with a focus on depressed mood as a transdiagnostic target across mental disorders. The paper has several strengths, notably the inclusion of clinicians and people with lived-experience of anxiety and/or psychosis in shaping the research questions and interpreting the findings, as well as the decision to broaden the date range of the search criteria (an acknowledgement that "transdiagnostic" concepts existed before "transdiagnostic' was named). There are also some considerations outlined below that form the basis for my recommendation for a "major revision".

- Major point: The paper would be strengthened by more theoretical discussion of the distinction between depressed mood and depression even though addressed in results and

discussion. Defined as “sub-threshold” depression – how to distinguish depressed mood as a transdiagnostic feature vs. comorbid depression (given high comorbidity)? This is mentioned in introduction (p. 6, lines 125 – 140) and in the “Diagnosis and Measurement” section on p.16, lines 325 – 342, but could be expanded further to reflect on the results of the current review, and the content of current measures, which would give way to a discussion of depressed mood as a transdiagnostic mechanism rather than merely a shared symptom. For example, a single item about depressed mood in a PANSS is quite different to the collection of 9-items in the PHQ-9, one of which is “feeling down, depressed, or hopeless”. The discussion would be improved with an expanded discussion about this observation – about whether depressed mood is “subthreshold depression” (including all DSM-5 symptoms) or just the depressed mood itself without associated symptoms (e.g., changes to sleep and appetite which also occur in anxiety and psychosis regardless of mood). Related to this point, it would be helpful to include a discussion of the relationship of depressed mood to anhedonia (another very similar transdiagnostic target: Guineau MG, Ikani N, Rinck M, Collard RM, van Eijndhoven P, Tendolkar I, Schene AH, Becker ES, Vrijsen JN. Anhedonia as a transdiagnostic symptom across psychological disorders: a network approach. Psychol Med. 2023 Jul;53(9):3908-3919. doi: 10.1017/S0033291722000575)

- It would be helpful to further clarify the inclusion of some articles when the focus is defined as the role of depressed mood in anxiety and/or psychosis e.g., p. 10, lines 238-241 – rationale for why pregnant women, people with diabetes students, epilepsy etc are included? Although this does strengthen argument for depressed mood as a transdiagnostic factor, the review is centred around depressed mood in anxiety and psychosis. Alternatively, could change title and framing to include “across disorders rather than just anxiety and psychosis.

- Rationale for different treatment interventions included? Possible mechanistic explanations as to why they may target depressed mood? Related to this, “CBT” is a broad umbrella – would be helpful to look at component parts (e.g., cognitive therapy, behavioural activation, positive affect interventions for anhedonia) to see differential effects on depressed mood.

- The research questions and aims differ slightly between the protocol and this paper. Please clarify what has changed and why (e.g., research questions in the protocol included a specific question about the effectiveness of interventions for depressed mood in anxiety and/or psychosis but this is not fully addressed in this paper).

- P.14, line 275: “Our review demonstrates tentative evidence regarding the positive effect of psychosis and anxiety interventions on depressed mood” – suggest amending this statement to include “interventions delivered to people experiencing psychosis and anxiety” as many of the interventions listed did not necessarily target psychosis and/or anxiety. For example, Laithwaite (2007) was a group-based self-esteem program delivered to people experiencing schizophrenia. In this study, it is likely that the self-esteem program positively influenced depressed mood, rather than anything specific to psychosis.

## VERSION 1 - AUTHOR RESPONSE

| **Reviewer Number** | **Original comments of the reviewer** | **Suggested Reply by the author(s)** | **Changes done on line number** |
|---|---|---|---|
| Reviewer 1 | The authors have conducted a scoping review to synthesise knowledge on depressed mood, its measurement, and interventions among persons with anxiety and/or psychosis.<br>The article is well written and the methodology of the review is stringent. I have some concerns that I would like the authors to address. | | |
| | The authors mention that to meet criteria for depressed mood the participants should not have clinically significant distress/dysfunction. However, they make no mention of how this was assessed in the studies included in the review or in their discussion. | *Thank you for the comment. Depressed mood criteria was assessed based on how it was described in the included articles. It was reported as depressed mood or with scores that were not sufficient for a clinical diagnosis.* | |
| | How was the data extraction form developed and was it trialed in any way? | Thank you for the question. The research team developed the data extraction form, through an iterative process, to capture bibliometrics and data related to the scoping review questions. It was tested on a small sample of articles and refined appropriately. | Page 8. Lines 203 - 206 |

| Reviewer Number | Original comments of the reviewer | Suggested Reply by the author(s) | Changes done on line number |
|---|---|---|---|
| | While not part of their scoping review they should discuss the concept of sub-threshold depression, which in many ways is better defined and how do they think depressed mood is different from it and the need to focus on depressed mood. Otherwise, we have various terminologies being used which are both overlapping and confusing. | Thank you very much for the suggestion. We discuss the concept of subthreshold depression, differentiating it from depressed mood which was the focus of this study. We acknowledge that depressed mood is also being used interchangeably with subthreshold depression. | Page 18; Lines 398-408 |
| | Was any ethical approval or consent sought for the inclusion of the panel comprising experts and those with a lived experience? | Thank you for the question. No ethical approvals were sought as stakeholders were consulted as collaborators and not as participants (Page 9, Lines 217-222); for feedback to refine the scoping reviews focus and feedback on the results for contextual relevance in a co-creation process. They are duly acknowledged for their role and immense contributions in the scoping review process (Page 22, Lines 547- 549). | |
| | | | |
| Reviewer 2 | Thank you for allowing me to review this manuscript. I believe that this is good research that pays attention to the depression or subthreshold depression that may affect the quality of life of patients with anxiety and psychosis. However, nowadays there is the word " anhedonia, or emotional bunting, is one of the more common depressive symptoms that may not have been included in the study. I want the author to discuss this in detail. | Thank you for the comment. The focus of our scoping review was depressed mood. However, we have included in the discussion how anhedonia and emotional blunting are related to depressed mood. | Page 18, Lines 408 - 419 |

| **Reviewer Number** | **Original comments of the reviewer** | **Suggested Reply by the author(s)** | **Changes done on line number** |
|---|---|---|---|
| | In addition, I would like the author to check the English language, including the uppercase or lowercase letters used in some points that may not be appropriate. Thanks, and good luck. | Thank you. We have reviewed the English and reserved in text upper case for proper nouns only. | |
| | | | |
| | | | |
| Reviewer 3 | Thank you for the opportunity to review this interesting paper, it is well-presented and outlines a thought-provoking scoping review. The rationale for the review is clear with a focus on depressed mood as a transdiagnostic target across mental disorders. The paper has several strengths, notably the inclusion of clinicians and people with lived-experience of anxiety and/or psychosis in shaping the research questions and interpreting the findings, as well as the decision to broaden the date range of the search criteria (an acknowledgement that "transdiagnostic" concepts existed before "transdiagnostic' was named). There are also some considerations outlined below that form the basis for my recommendation for a "major revision". | | |

| | | | |
|---|---|---|---|
| | Major point: The paper would be strengthened by more theoretical discussion of the distinction between depressed mood and depression even though addressed in results and discussion. Defined as “sub-threshold” depression – how to distinguish depressed mood as a transdiagnostic feature vs. comorbid depression (given high comorbidity)? This is mentioned in introduction (p. 6, lines 125 – 140) and in the “Diagnosis and Measurement” section on p.16, lines 325 – 342, but could be expanded further to reflect on the results of the current review, and the content of current measures, which would give way to a discussion of depressed mood as a transdiagnostic mechanism rather than merely a shared symptom. For example, a single item about depressed mood in a PANSS is quite different to the collection of 9-items in the PHQ-9, one of which is “feeling down, depressed, or hopeless”. The discussion would be improved with an expanded discussion about this observation – about whether depressed mood is “subthreshold depression” (including all DSM-5 symptoms) or just the depressed mood itself without associated symptoms (e.g., changes to sleep and appetite which also occur in anxiety and psychosis regardless of mood). Related to this point, it would be helpful to include a discussion of the relationship of depressed mood to anhedonia (another very similar transdiagnostic target: Guineau MG, Ikani N, Rinck M, Collard RM, van Eijndhoven P, Tendolkar I, Schene AH, Becker ES, Vrijsen JN. Anhedonia as a transdiagnostic symptom across psychological disorders: a network approach. Psychol Med. 2023 Jul;53(9):3908-3919. doi: 10.1017/S0033291722000575) | Thank you very much for the suggestions. We have expanded our discussion of depressed mood vs. depression or subthreshold depression. The focus of this paper was on depressed mood as a transdiagnostic target in psychosis/ anxiety.<br><br>We have included a discussion on the psychometric robustness of the various tools used to measure depressed mood comparing the single item screening of the PANSS to the multiple item screening of the PHQ-9 and BDI.<br><br>We have also acknowledged that this area needs further conceptualisations to reduce the overlapping constructs and improve the clinical utility of the concepts.<br><br>We have also include a discussion on anhedonia in relation to depressed mood. In our conceptualisation of depressed mood, we have acknowledged anhedonia as one of its characterisations which may be present or absent, where it does not lead to | Page 18; Lines 398 – 408<br><br>Page 19; Lines 461 – 466<br><br>Page 18. Lines 420 – 425<br><br>Page 18; Lines 408 - 413 |

| Reviewer Number | Original comments of the reviewer | Suggested Reply by the author(s) | Changes done on line number |
|---|---|---|---|
| | | clinically significant impairment | |
| | It would be helpful to further clarify the inclusion of some articles when the focus is defined as the role of depressed mood in anxiety and/or psychosis e.g., p. 10, lines 238-241 – rationale for why pregnant women, people with diabetes students, epilepsy etc are included? Although this does strengthen argument for depressed mood as a transdiagnostic factor, the review is centred around depressed mood in anxiety and psychosis. Alternatively, could change title and framing to include "across disorders rather than just anxiety and psychosis. | Thank you. The focus of the review was depressed mood in anxiety and/or psychosis. Anxiety and/or psychosis was reported in the studies as co-morbid with the main conditions, and depressed mood also assessed. However, we acknowledge the limitation of our study in that co-morbidity introduced confounders that could have also influenced the reported findings | Page 4, Lines 89 - 91 |
| | Rationale for different treatment interventions included? Possible mechanistic explanations as to why they may target depressed mood? Related to this, "CBT" is a broad umbrella – would be helpful to look at component parts (e.g., cognitive therapy, behavioural activation, positive affect interventions for anhedonia) to see differential effects on depressed mood. | Thank you for this suggestion. That would be a strength going forward. However, in the current study the included articles did not give a breakdown of the CBT interventions. This is acknowledged as a limitation of the current study because of the nature of the studies reported. | Page 14; Lines 285 - 290 |

| Reviewer Number | Original comments of the reviewer | Suggested Reply by the author(s) | Changes done on line number |
|---|---|---|---|
| | The research questions and aims differ slightly between the protocol and this paper. Please clarify what has changed and why (e.g., research questions in the protocol included a specific question about the effectiveness of interventions for depressed mood in anxiety and/or psychosis but this is not fully addressed in this paper). | Thank you for this question. The current scoping review aims are reported in alignment with the quality of the papers that were reviewed. Some of the original research questions could not be answered by the quality of the papers included and hence the questions were modified accordingly as is permissible for a scoping review. Although it would be good to assess the effectiveness of the interventions, the identified article do not permit for such an assessment, and that might need a systematic review with sufficient data to answer that question. | Page 21, Lines 502 - 508 |
| | P.14, line 275: "Our review demonstrates tentative evidence regarding the positive effect of psychosis and anxiety interventions on depressed mood" – suggest amending this statement to include "interventions delivered to people experiencing psychosis and anxiety" as many of the interventions listed did not necessarily target psychosis and/or anxiety. For example, Laithwaite (2007) was a group-based self-esteem program delivered to people experiencing schizophrenia. In this study, it is likely that the self-esteem program positively influenced depressed mood, rather than anything specific to psychosis. | Thank you for the suggestion. We have revised the sentence. It now reads. "Our review demonstrates tentative evidence regarding the positive effect of interventions delivered to people experiencing psychosis and anxiety on depressed mood." | Page 14, Lines 281-282 |

**VERSION 2 - REVIEW**

**Reviewer** 3

**Name** Black, Melissa

**Affiliation** MRC Cognition and Brain Sciences Unit, University of Cambridge

**Date** 26-Oct-2025

**COI**

The authors have addressed all my previous comments. Congratulations on a geat paper.
